# The “Janus” Role of C/EBPs Family Members in Cancer Progression

**DOI:** 10.3390/ijms21124308

**Published:** 2020-06-17

**Authors:** Manlio Tolomeo, Stefania Grimaudo

**Affiliations:** Department of Health Promotion Sciences, Maternal and Infant Care, Internal Medicine and Medical Specialties, University of Palermo, via del Vespro 129, 90127 Palermo, Italy; stefania.grimaudo@unipa.it

**Keywords:** C/EBP, cancer, tumor promoter, tumor suppressor

## Abstract

CCAAT/enhancer-binding proteins (C/EBPs) constitute a family of transcription factors composed of six members that are critical for normal cellular differentiation in a variety of tissues. They promote the expression of genes through interaction with their promoters. Moreover, they have a key role in regulating cellular proliferation through interaction with cell cycle proteins. C/EBPs are considered to be tumor suppressor factors due to their ability to arrest cell growth (contributing to the terminal differentiation of several cell types) and for their role in cellular response to DNA damage, nutrient deprivation, hypoxia, and genotoxic agents. However, C/EBPs can elicit completely opposite effects on cell proliferation and cancer development and they have been described as both tumor promoters and tumor suppressors. This “Janus” role of C/EBPs depends on different factors, such as the type of tumor, the isoform/s expressed in cells, the type of dimerization (homo- or heterodimerization), the presence of inhibitory elements, and the ability to inhibit the expression of other tumor suppressors. In this review, we discuss the implication of the C/EBPs family in cancer, focusing on the molecular aspects that make these transcription factors tumor promoters or tumor suppressors.

## 1. Introduction

CCAAT-enhancer-binding proteins (C/EBPs) is a family of six structurally homologous transcription factors that promote the expression of genes involved in different cellular responses, such as proliferation, growth, and differentiation. These transcription factors control the differentiation of several cell types, and have key roles in regulating cellular proliferation, through interaction with cell cycle proteins. The molecular structure of C/EBPs and their ability to interact with a multitude of factors determine their complex functions in different cells. In fact, C/EBPs can be activated or inhibited by a variety of intracellular or extracellular signals. In addition, post-translational modifications and interaction with other proteins can regulate their expression and activity in a complex manner [1]. C/EBPs can activate or repress several classes of genes implicated in cell differentiation, metabolism, inflammation, and immune response. Moreover, C/EBPs play an important role in cancer progression and metastasis, showing both pro-oncogenic and onco-suppressor functions. Interestingly, the same isotype of C/EBP can exhibit both of these opposite functions. This “Janus” role of C/EBPs in cancer could depend on their particular position at the crossroads between proliferation and differentiation. Specific conditions such as cell type, microenvironment, type of heterodimerization, or interaction with different regulatory proteins can tip the balance towards pro- or anti-oncogenic action [2].

## 2. C/EBPs Structure and Isoforms

C/EBPs are constituted by different functional and structural components, which include a C-terminal leucine-zipper (a basic DNA-binding region) and in the N-termini of most C/EBP proteins (regions that act as transactivating and regulatory domains).

The leucine zipper is a highly conserved protein segment with a periodic repetition of leucine residues at every seventh position over a distance covering eight helical turns. The polypeptide segments containing these periodic arrays of leucine residues form a continuous α-helix that can dimerize through formation of a coiled-coil structure involving paired contacts between hydrophobic leucine zipper domains. Dimerization through the leucine-zipper leads to formation of homo- and heterodimers, which then bind with their two basic regions to DNA-sequences in the promoter/enhancer regions of a variety of genes. The basic residues interact in the major groove of the DNA, forming sequence-specific interactions [3].

The dimerization and the localization of leucines are critical for the DNA binding to the basic region. The basic regions of C/EBPs show a high binding affinity for (G/A)TTGCG(T/C)AA(T/C) or, broadly, the promoter CCAAT box sequence [4]. The N-termini of the C/EBP proteins are quite divergent, except for three short sub-regions that are conserved in most members and that represent the activation domains. The N-termini of some C/EBP proteins also contain regulatory domains that are conserved in most members [5]. Once bound to DNA, C/EBPs can recruit co-activators in their activation domains that, in turn, can open up a chromatin structure or recruit basal transcription factors that stimulate transcription.

**Figure 1:**
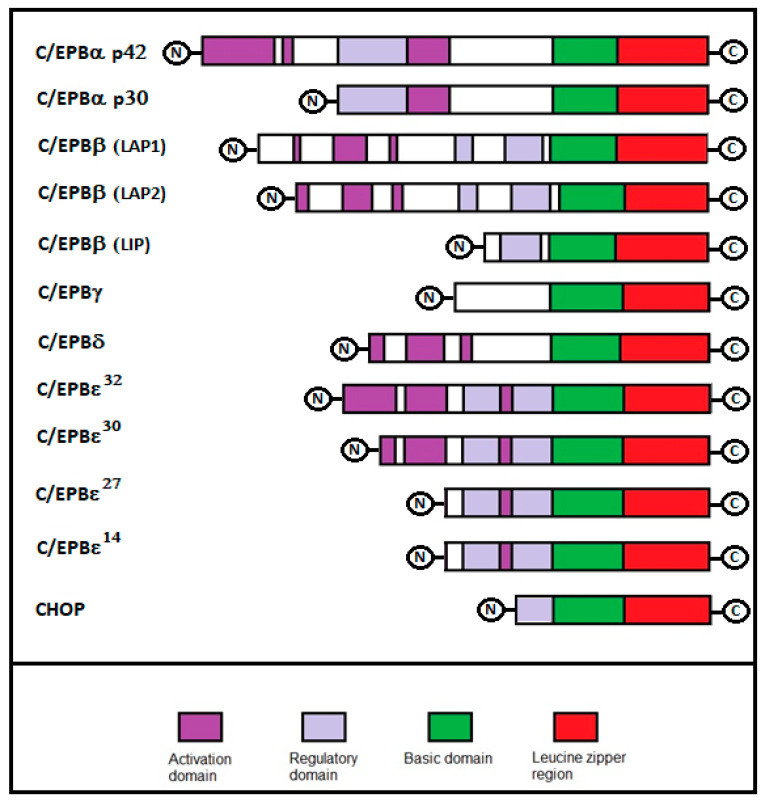
Structure of the C/EBP transcription factor family. Each member of this family of proteins share a high degree of sequence similarity at the C-terminus, including the leucine zipper domain and the basic region. At the N-terminus, the structures vary among different family members and among different isoforms.

The C/EBP family consists of six structurally and functionally homologous transcription factors—C/EBPα, C/EBPβ, C/EBPδ, C/EBPγ, C/EBPε, and CHOP (Figure 1). Similarities between C/EBP family members suggest an evolutionary history of genetic duplications with a subsequent pressure to diversify them. Each member of this family varies in tissue specificity and transactivating activity. The genes encoding C/EBPβ, C/EBPδ, C/EBPε, and CHOP have been mapped on chromosome 20, 8, 14, and 12 respectively. C/EBPα and C/EBPγ are both located on chromosome 19. However, despite the existence of six genes, the number of C/EBP proteins can be considerably higher. This depends on the production of different sized C/EBP proteins, due to the alternative use of translation initiation codons in the same mRNA molecule, and the alternative use of promoters or differential splicing [6,7,8]. Moreover, for some members, the expression of the protein does not correlate with that for mRNA, indicating tissue-specific post-transcriptional regulatory mechanisms. Apart from C/EBPγ and CHOP that are expressed ubiquitously, the other isoforms of C/EBP are distributed in specific tissues. For example, C/EBPα is prevalently expressed in the adipose tissue, blood mononuclear cells, liver, intestine, lung, adrenal gland, blood, nervous system, and placenta; C/EBPβ is expressed in the liver, adipose tissue, myelomonocytic cells, intestine, lung, spleen, nervous system, and kidney; C/EBPδ in the adipose tissue, myeloid cells, lung, nervous system, and intestine; and C/EBPε is expressed in myeloid and lymphoid cells (Table 1). The importance of the C/EBP family in adipose tissues was confirmed by numerous studies that have shown the role of C/EBPα, β, and γ in adipocyte differentiation [9,10,11,12]. Four members of the C/EBP family (α, β, δ, and ε) are expressed in myeloid cells where they play a crucial role in myeloid-cell differentiation [13,14,15]. C/EBPα and C/EBPβ are widely expressed in hepatocytes, where they are involved in the regulation of terminal hepatocyte differentiation and in the maintenance of normal function and responses to injury [16]. CEBPβ is required for mammary-gland development, for the functional differentiation of mammary epithelial cells and the expression of milk protein genes [17]. Finally, CEBPs are implicated in the differentiation of keratinocytes (C/EBPβ), neuronal cells (C/EBPβ), and intestinal epithelial cells (C/EBPα) [18,19,20].

## 3. C/EBPs Functions

C/EBPα plays a main role during the late phases of differentiation of pre-adipocytes. Both C/EBPβ and C/EBPδ mRNA are induced during the mitotic expansion of pre-adipocite cells. When pre-adipocytes exit, the cell cycle begins to express C/EBPα, which is then followed by the induction of adipocyte-specific markers [21]. It seems that C/EBPα expression is activated by the binding of C/EBPβ and C/EBPδ, with the C/EBPα promoter. C/EBPs are also implicated in myeloid differentiation [1]. Binding sites for the C/EBPs (α, β, δ, and ε) are present in the promoter regions of numerous genes that are expressed in myeloid cells. The expression of C/EBPα is relatively high in early myeloid progenitors and decreases during granulocytic differentiation. On the other hand, C/EBPε, is preferentially expressed during granulocytic differentiation, whereas C/EBPβ is up-regulated during macrophage differentiation. C/EBP proteins also have a role in the differentiation of hepatocytes, mammary, epithelial cells, ovarian luteal cells, keratinocytes, neuronal cells, and intestinal epithelial cells [1]. C/EBPs are implicated in the control of metabolism and in inflammation, as shown by the identification of binding sites for the C/EBPs, in the regulatory regions of a battery of genes involved in the inflammatory response, including those coding for cytokines and their receptors, acute-phase plasma proteins, and components of signal transduction pathways. Moreover, C/EBPα, C/EBPβ, and C/EBPδ were shown to be widely expressed in the mammalian nervous system and these seemed to be involved in the memory process [22]. Finally, most members of the C/EBPs family are implicated in the control of cell cycle and are involved during cancer progression, showing both tumor promoter and tumor suppressor activities.

## 4. C/EBPs and Cancer

C/EBPs are considered tumor suppressor factors for their ability to block cell growth, and for their role in cellular response to DNA damage. However, C/EBPs can elicit completely opposite effects on cell proliferation and cancer development, depending on the cell-type and the isoform present. They have also been described as both tumor promoters and tumor suppressors. This “Janus” role has been observed for all members of the C/EBP family (Table 2).

### 4.1. C/EBPα

C/EBPα was the first member of C/EBP family cloned. It is expressed prevalently in post-mitotic cells and it seems implicated in regulation of cell-cycle exit and differentiation in adipocytes, hepatocyte, myeloid cells, and other tissues [11,13,16,20,23]. C/EBPα induces cell growth arrest by blocking the association of CDKs with cyclins [24] and stabilizing the CDK2-p21 inhibitory complex [25]; moreover, C/EBPα can directly inhibit the activity of free CDK2/CDK4 [24]. In addition, C/EBPα might also associate with E2F complexes and convert them into repressors capable of inhibiting the S-phase gene transcription [26]. In this context, C/EBPα plays a role in the cellular response to DNA damage induced by extrinsic DNA-damaging agents. In fact, the C/EBPα gene is a p53-regulated DNA damage-inducible gene in keratinocytes and it is an important link between UVB-induced DNA damage and cell cycle arrest in epidermal keratinocytes [27].

Two isoforms of the C/EBPα are generated from its mRNA by a ribosomal scanning mechanism—the full-length 42 kDa C/EBPα (p42), which is implicated in the transcriptional activation of adipocyte genes and the 30-kDa isoform (p30). p30 is an alternative translation product initiated at the third in-frame methionine codon of the C/EBPα mRNA. Unlike p42, which inhibits cell proliferation, p30 seems not to exert an antiproliferative function [28].

For its ability to induce growth arrest contributing to the terminal differentiation of several cell types, and for its role in the cellular response to DNA damage, C/EBPα is considered a potent tumor suppressor factor. Deregulation of its expression can predispose to different malignancies, especially hematological neoplasms.

In fact, C/EBPα plays a main role in hematopoiesis. It is essential for myeloid differentiation and it has been implicated in regulating self-renewal of fetal liver and adult hematopoietic stem cells. Disruption of C/EBPα blocks the transition from myeloid committed stem cells to granulocyte/monocyte progenitors, leading to the loss of mature granulocytes [29]. In adult hematopoietic stem cells, the loss of C/EBPα causes increased proliferation, an increased number of functional long-term hematopoietic stem cells, and advanced repopulating ability [30]. N-Myc seems to be the downstream target of C/EBPα in hematopoietic stem cells. In fact, transcriptional repression of N-Myc by C/EBPα maintains the hematopoietic stem cells in a quiescence status [31]. Downregulation of C/EBPα plays a role in leukemogenesis. C/EBPα function is indeed frequently abrogated in acute myelogenous leukemias (AML) and oncogenes, such as AML1-ETO, BCR-ABL, or FLT3-ITD. This can downregulate or suppress C/EBPα, causing a block in myeloid differentiation and thereby inducing leukemogenesis [32,33,34,35]. Deregulation of the C/EBPα expression was also reported in a variety of additional human tumors, including breast and lung cancer [36].

Discordant results were observed in hepatocellular carcinoma (HCC) and in hepatoblastoma (HBL). Tomizawa et al. examined the expression level of the C/EBPα and C/EBPβ genes between tumor and non-tumorous tissues of the same hepatocellular carcinoma patients, with quantitative real-time polymerase chain reactions showing that the expression of both the C/EBPα and C C/EBPβ genes was downregulated in the majority of the tumor specimens compared to the corresponding non-tumorous tissues. Patients whose expression of either C/EBPα or C/EBPβ was higher in tumors than non-tumorous tissues, survived longer than those whose expression was lower in tumors [37]. Similar results were obtained by Tseng et al. in a retrospective cohort study on 50 HCC patients. They observed that a reduced expression of the C/EBPα protein in HCC was associated with an advanced tumor stage and shortened patient survival [38]. In contrast, Lu et al. demonstrated that C/EBPα overexpression was correlated with poorer HCC overall survival [39]. Moreover, upregulation of C/EBPα was described in hepatoblastoma (HBL) [40]. These data suggest that C/EBPα might act as both a tumor suppressor and tumor promoter factor in liver cancers. These opposite functions might be correlated to a different posttranslational phosphorylation switch of C/EBPα. Phosphorylation of C/EBPα at ser190 (ser193 in mice homologue) is essential to maintain quiescence of hepatocytes through two pathways—inhibition of cdks and repression of E2F [41]. However, in liver tumor cells, the activation of the PI3K/Akt pathway blocks the growth inhibitory activity of C/EBPα, through the PI3K/AKT-protein phosphatase 2 (PP2A)-mediated dephosphorylation of C/EBPα on Ser 193 [41]. Dephosphorylated C/EBPα is unable to interact with and inhibit cdks and E2F with the consequent promotion of cell growth. Mutation of Ser 193 to Ala also abolishes the ability of C/EBPα to cause growth arrest, as it prevents the interaction of C/EBPα with cdk2 and E2F-Rb complexes [41]. Cast et al. investigated liver cancer in the mouse model C/EBPα-S193A, in a large cohort of human HBL samples, and in Pten/p53 double knockout mice, and found that these cancers were characterized by an elevation of C/EBPα, which was dephosphorylated at Ser190/193. They found that dephosphorylated C/EBPα creates preneoplastic foci with cancer stem cells that give rise to HCC and aggressive HBL [42]. Therefore, conversion of the tumor suppressor C/EBPα into an oncogenic isoform can create preneoplastic foci where hepatocytes dedifferentiate into cancer cells, giving rise to liver cancer expressing high levels of mutated C/EBPα.

The development of liver cancer can also be determined by degradation of C/EBPα. Carcinogens such as diethylnitrosamine/phenobarbital (DEN/PB) can induce specific degradation of the phosphorylated isoform of C/EBPα, through activation of the ubiquitinproteasome system (UPS). The mechanism of the UPS-mediated elimination of C/EBPα during carcinogenesis involves elevated levels of gankyrin (an oncogenic protein that was found to interact with the phosphorylated form of C/EBPα) and targets it for UPS-mediated degradation [43,44,45].

### 4.2. C/EBPβ

C/EBPβ, which initially denominated the nuclear factor for IL-6 (NF-IL6), was first described in 1990 as a factor binding to the interleukin 1 (IL-1)-responsive element in the IL-6 promoter and showed high C-terminal homology to C/EBPα [46]. Subsequent knockout experiments revealed that the C/EBPβ knockout mice were viable but exhibited female sterility, defective mammary epithelial differentiation, and impaired immune function [1].

In fact, C/EBPβ is implicated in cell differentiation and in the regulation of genes involved in immune and inflammatory responses, such as *IL-6*, *IL-4*, *IL-5*, and *TNF-alpha* genes [15,47,48,49]. Moreover, it is critical for macrophage and B-cell differentiation [50,51]. C/EBPβ can activate genes that have specific roles in the nervous system—it can interact with an element of the preprotachykinin-A promoter, facilitating the substance P precursor gene transcription and with the promoter P2 of the choline acetyltransferase gene, inducing the biosynthesis of acetylcholine [52]. C/EBPβ also seems to be implicated in activation of genes coding for proteins that confer multidrug resistance to the cells regulating the liver expression of the MRP2 gene and activating the MDR1 gene in the MCF-7 cells [53,54].

The implication of C/EBPβ in cancer and tumorigenesis is more complex than that of C/EBPα. Many biological properties of C/EBPβ are similar to those of C/EBPα (since it blocks proliferation, promotes differentiation, and suppresses tumorigenesis), and similarly to C/EBPα. C/EBPβ is able to suppress cell proliferation through repression of the E2F target genes, thereby causing cellular senescence [55].

Expression of oncoproteins in primary cells often provokes cellular senescence, which is a permanent state of cell growth arrest that acts as a tumor suppressor mechanism. This cytostatic response, termed oncogene-induced senescence (OIS) is implemented through induction of the p19Arf-p53 tumor suppressor pathway and CDK inhibitors, such as p16Ink4a and p21CIP1, which activate Rb-dependent checkpoints [56,57,58]. C/EBPβ exerts anti-oncogenic effects because it is required for OIS.

Nevertheless, C/EBPβ can also exert pro-oncogenic effects [59]. These opposite activities depend on different causes, such as homo- or -heterodimerization, presence of inhibitors or presence of different isoforms of C/EBPβ.

C/EBPβ is maintained in a latent state by several auto-inhibitory elements that suppress its DNA-binding and transactivation functions [5,60]. Oncogenic stimuli, such as oncogenic RAS, can activate the RAF–MEK–ERK pathway that causes the phosphorylation and activation of the C/EBPβ [60,61]. Oncogenic Ras also increases the ratio of C/EBPβ homodimers with respect to C/EBPβ:C/EBPγ heterodimers, through a mechanism involving phosphorylation on leucine zipper residue Ser273 by p90Rsk kinases [62]. The homodimeric form of C/EBPβ contributes to the Ras-induced cell-cycle arrest and senescence in primary cells, whereas β:γ heterodimers actively promote cell growth [62]. Moreover, in immortalized and transformed cells, Ras-induced post-translational activation of C/EBPβ is inhibited by the 3′ untranslated region (3′UTR) of its mRNA, suppressing the cytostatic and pro-senescence functions of C/EBPβ [63]. The 3′UTR inhibitory effect was mapped to a region bearing the G/U rich elements (GREs). Moreover, an AU-rich element (ARE) and the ARE/GRE-binding protein HuR are required for 3′UTR inhibition. These components act by directing C/EBPβ transcripts to the peripheral cytoplasm, excluding them from a perinuclear region where the C/EBPβ kinases ERK1/2 and CK2 reside in RAS-transformed cells. In this location, newly translated C/EBPβ is uncoupled from RAS signaling and fails to undergo phosphorylation and activation by the RAF–MEK–ERK pathway. Thus, the intracellular site of the C/EBPβ translation is critical for RAS-induced activation via effector kinases such as ERK. Interestingly, 3′UTR inhibition and C/EBPβ mRNA compartmentalization are not observed in primary mouse and human fibroblasts [64].

The pro- and anti-tumorigenic activities of C/EBPβ can be, in part, determined by the presence of different isoforms of C/EBPβ. In fact, C/EBPβ is expressed as three isoforms with distinct activities—full-length LAP1 (or C/EBPβ p38 or Liver Activating Protein* (LAP*)), LAP2 (or C/EBP-β p32) that are lacking 21 amino acids (23 in human proteins) from the N-terminus, and Liver Inhibitory Protein (LIP or C/EBPβ p20) that lacks the whole activation domain (TAD) and acts as a transcriptional repressor. LAP1 is generally involved in terminal differentiation of cells, whereas LAP2 and LIP promote cell proliferation and tumor progression [65]. The proper ratios of the three isoforms are critical for normal cell growth and development. For example, an excess of the LIP isoform was observed in tumor cells that evade the growth inhibitory action of TGFβ. In normal cells, TGF-β block the cell cycle at the G1 phase, induce differentiation, or promote apoptosis. C/EBPβ is essential for TGFβ induction of the cell cycle inhibitor p15 INK4b and repression of C-Myc in human epithelial cells. These cytostatic responses can be missing in metastatic breast cancer cells. This loss seems to depend on the excess of the C/EBPβ inhibitory isoform LIP [66]. An excess of LIP was also observed in FLT3-ITD signal transduction. FLT3 is a cytokine receptor involved in cell growth and apoptosis regulation. Mutations that cause constitutive activation of the FLT3 receptor are frequent in acute myelogenous leukemia (AML) patients. The most frequent FLT3-mutations in AML are internal tandem duplications (ITDs) that lead to a constitutive activation of this receptor. In FLT3-ITD positive cells or when ITD sequences were inserted into the FLT3-wild type receptor, the LIP and LIP/LAP ratios were significantly increased, showing enhanced proliferation rates of AML cells. In addition, incubation of the FLT3-wild type cells with the FLT3 receptor ligand also elevated the LIP, LIP/LAP ratios, and proliferation [67].

### 4.3. C/EBPδ

C/EBPδ is a transcription factor that modulates many biological processes including cell growth, differentiation, motility, and cell death. To date, only one full-length protein isoform is known. The C/EBPδ gene is induced during growth arrest and cell differentiation. For example, C/EBPδ regulates growth arrest (G0–G1) and apoptosis in mammary epithelial cells, and promotes contact inhibition in these cells [68,69] and in fibroblasts [70]. Moreover, it mediates the growth arrest of mammary epithelial cell lines, in response to oncostatin M [71]. Due to its roles in cell-growth arrest, cell differentiation, and apoptosis, C/EBPδ acts as a tumor suppressor especially in the early stages of tumor development. Moreover, C/EBPδ is also implicated in the regulation of gene transcription of several inflammatory cytokines and acute-phase proteins [72].

The antiproliferative effect of C/EBPδ seems to depend on its ability to downregulate the expression of cyclin D/E and to upregulate the expression of the cyclin-dependent kinase inhibitor p27CIP2. These effects prevent the cyclin-dependent phosphorylation of the retinoblastoma protein (Rb) and the G1-S transition [68,73]. For these reasons, C/EBPδ acts as a potent tumor-suppressor factor and is frequently downexpressed in different malignancies. For example, the C/EBPδ promoter is silenced in about 35% of human acute myeloid leukemia samples [74], and its expression is downregulated in the blast crisis phases of chronic myelogenous leukemia (CML) [75]. Of interest, experimental induction of C/EBPδ in BCR-ABL-positive CML blast cells resulted in G0/G1 proliferative arrest, and a moderate increase in apoptosis. C/EBPδ also plays a role also in cell differentiation, as shown by myeloid differentiation of the CML blast cells after C/EBPδ induction [76].

Overexpression of C/EBPδ leads to myeloid differentiation accompanied by upregulation of the G-CSF receptor and downregulation of C-Myc expression, also in primary mouse hematopoietic progenitor cells or AML cell lines [74,77]. Indeed, C/EBPδ is expressed in differentiated granulocytes [13,76]. In macrophages, C/EBPδ regulates many genes associated with functions of differentiated cells and is specifically associated with M1 macrophage polarization [78].

A significant correlation was observed between hypermethylation of the C/EBPδ promoter and low expression of C/EBPδ in AML patients, indicating that this tumor suppressor gene can be silenced by promoter methylation [74].

C/EBPδ is induced by 1,25(OH)2D3 in several cancer cells, including estrogen receptor (ER)-expressing breast cancer cells that are sensitive to the growth inhibitory effects of 1,25(OH)2D3. However, 1,25(OH)2D3 is not able to induce C/EBPδ in either androgen receptor-negative or ER-negative breast cancer cells that are relatively resistant to growth inhibition by 1,25(OH)2D3. Of interest, forced expression of C/EBPδ in 1,25(OH)2D3 resistant cells dramatically reduces their clonal growth, suggesting that forced expression of C/EBPδ in cancer cells might be an effective therapeutic strategy [79].

However, C/EBPδ does not necessarily inhibit cell proliferation and can be compatible with cell proliferation. In fact, C/EBPδ promotes the proliferation of cultured osteoblasts by directly activating the expression of insulin-like growth factor-1 (IGF-1) [80] and promotes the proliferation of vascular smooth muscle cells in response to IL-1β, by inducing expression of the PDGFA-receptor [81]. In a mouse model of mammary tumorigenesis, C/EBPδ reduces tumor incidence but promotes tumor metastasis. That is, the encoding of the F-box protein FBXW7 that promotes degradation of the mammalian target of rapamycin (mTOR), at least in part, depends on the ability of C/EBPδ to directly inhibit the expression of the tumor suppressor *F-box* and *WD repeat-domain containing 7* gene (*FBXW7*). Consequently, C/EBPδ enhances the mTOR/AKT/S6K1 signaling and augments translation and activity of hypoxia-inducible factor-1α (HIF-1α), which is necessary for hypoxia adaptation of metastasis [82].

C/EBPδ is a tumor promoter in brain cancer. Indeed, C/EBPδ mRNA is overexpressed in mesenchymal glioblastoma cells and is associated with poor prognosis [83,84]. This could be due to an overexpression of HIF-1α induced by C/EBPδ, which allows the survival of tumor cells under hypoxic conditions. Furthermore, C/EBPδ inhibits the expression of FBXW7α in glioblastoma cell lines, thus, contributing to glioblastoma progression [82,85].

Finally, C/EBPδ mRNA can be overexpressed in cancer cells expressing high levels of STAT3. The C/EBPδ gene promoter can be activated by the STAT3 transcription factor [86], which is frequently hyperactivated in cancer and is a well-characterized tumor-promoting factor [87]. Therefore, there are human breast cancer cells in which both STAT3 and the mRNA of the cancer suppressor C/EBPδ are overexpressed. STAT3 can be activated by the tyrosine kinase Src [87,88]. Src downregulates C/EBPδ through the SIAH2 E3 ubiquitin ligase. In particular, Src phosphorylates SIAH2 in vitro and leads to tyrosine phosphorylation and activation of SIAH2 in breast tumor cell lines. SIAH2 interacts with the C/EBPδ, promoting its polyubiquitination and proteasomal degradation. Src/SIAH2-mediated inhibition of C/EBPδ expression supports elevated cyclin D1 levels, phosphorylation of Rb, motility, invasive properties, and survival of the transformed cells. Src kinase activity downregulates C/EBPδ protein but not the mRNA levels [89]. Therefore, tumors overexpressing STAT3 can also express high levels of C/EBPδ mRNA but show low levels of C/EBPδ.

### 4.4. C/EBPγ

C/EBPγ was first identified by its affinity for cis-regulatory sites in the Ig heavy-chain promoter and enhancer. This member of C/EBPs family contains a C-terminal leucine-zipper but lacks an amino-terminal transactivation domain present in the other C/EBPs members. Transfection assays have shown that C/EBPγ is neither an activator nor a repressor of transcription but acts as a transdominant negative regulator of other C/EBPs [90]. C/EBPγ can repress the transcriptional activity of C/EBPβ, C/EBPα, and C/EBPδ, through heterodimerization with its partner. However, heterodimerization with C/EBPγ did not alter the DNA-binding specificity of its C/EBP partner, since both homodimers and heterodimers can efficiently bind to a consensus C/EBP element.

C/EBPγ-deficient mice showed a high mortality rate within 48 h after birth. C/EBPγ (−/−) mice showed normal T and B cell development. However, cytolytic functions of their splenic natural killer (NK) cells after stimulation with cytokines such as interleukin (IL)-12, IL-18, and IL-2 were significantly reduced and the ability of splenocytes of C/EBPγ (−/−) mice to produce interferon (IFN)-γ in response to IL-12 or IL-18 was markedly impaired [91].

An interesting function of C/EBPγ is its ability to inhibit cellular senescence. C/EBPγ (−/−) mouse embryonic fibroblasts and hematopoietic progenitors proliferated poorly and entered senescence prematurely. The senescence-suppressing activity of C/EBPγ requires its ability to heterodimerize with C/EBPβ. It was shown that the C/EBPβ depletion partially restored the growth of C/EBPγ-deficient cells, thus indicating that the increased levels of C/EBPβ homodimers in C/EBPγ (−/−) cells and inhibiting proliferation. Furthermore, high C/EBPγ expression correlated with poor clinical prognoses in several human cancers, and C/EBPγ depletion decreased proliferation and induced senescence in lung tumor cells [92].

C/EBPγ can have opposite functions depending on cell type and tissue. For example, as opposed to fibroblasts (L cells), C/EBPγ did not repress transactivation by C/EBPβ or C/EBPδ in HepG2 hepatoma cells, despite a lack of difference in heterodimer formation in HepG2 and L cells [93]. It was supposed that the presence of a pool of homodimers resistant to heterodimerization in HepG2 cells, perhaps because of a specific post-translational modification, could be the cause of the lack of C/EBPγ repression activity. However, because the amount of these homodimers is similar in L fibroblasts and HepG2, their presence cannot account for the differential repression by C/EBPγ. Another potential explanation for this difference in activity is that heterodimers could be the target of activating kinases in HepG2 cells but not in L cells, whereas homodimers might be effective substrates in both cells. Such modifications could occur on either the C/EBP activator protein or the C/EBPγ subunit. C/EBP proteins generally induce cell growth arrest but heterodimerization with C/EBPγ can mitigate the growth arrest activity of these proteins. However, C/EBPγ is unable to suppress C/EBP-mediated growth arrest in hepatoma cells, similar to its inability to inhibit C/EBP-dependent transcription in these cells. Finally, C/EBPα was not effectively inhibited by C/EBPγ in either L cells or HepG2 cells. Therefore, the ability of C/EBPγ to inhibit C/EBP activity is cell-specific and differs for the various C/EBP family members [93].

### 4.5. C/EBPε

C/EBPε is expressed exclusively in myeloid cells. C/EBP epsilon-deficient mice developed normally but failed to generate functional neutrophils and eosinophils, and these mice died of opportunistic infections suggesting that C/EBPε might play a central role in myeloid differentiation. It has an important role in the transcriptional regulation of a subset of myeloid-specific genes, by utilizing both activation and repression functions. In humans, C/EBPε is expressed as 4 distinct isoforms expressed as proteins of 32, 30, 27, and 14 kDa, through alternative splicing, differential promoter usage, and translational start sites [94,95]. They are required for the terminal differentiation of neutrophils and eosinophils and the highest levels of expression occur during the transition from promyelocyte to myelocyte cells. However, these isoforms can have opposite functions. The C/EBPϵ^32^ and C/EBPε^30^ isoforms are transcriptional activators, whereas C/EBPε^27^ is an inhibitor of GATA-1, a factor essential for the development of hematopoietic cells. C/EBPε^14^ contains only DNA-binding and DNA-dimerization domains and seems to act as a dominant-negative regulator.

C/EBPε^32^ and C/EBPε^30^ transduction of hematopoietic progenitor cells exclusively cause eosinophil differentiation. In contrast, the putative repressor C/EBPε^27^ isoform strongly inhibits eosinophil differentiation and gene expression, including GATA-1, promoting granulocyte (neutrophil)-macrophage differentiation. The C/EBPε^27^ repressor isoform strongly inhibits eosinophil development but promotes erythroid differentiation [96]. Therefore, by acting as an activator or repressor, C/EBPϵ isoforms can reprogram myeloid lineage commitment and differentiation. Although the allelic loss of the C/EBPε gene was detected in some acute myelogenous leukemias (AML) and in myelodysplastic syndrome (MDS) evolving to AML, there are no data concerning a specific tumor suppressor or tumor promoter function of C/EBPε [97].

### 4.6. CHOP

C/EBP homologous protein (CHOP), also known as growth arrest and DNA damage-inducible protein 153 (GADD153), is another member of the C/EBPs family that displays both tumor-suppressive or tumor-supporting roles. It plays a central role in endoplasmic reticulum (ER) stress, especially in cancer ER stress [98]. It is a stress-responsive transcription factor during growth arrest, DNA damage, nutrient deprivation, hypoxia, and genotoxic agents. CHOP controls numerous genes involved in different cellular processes including inflammation, differentiation, autophagy, and apoptosis.

CHOP exerts an anti-oncogenic function by activating apoptosis in damaged, mutated, or precancerous cells by modulating the expression of pro-apoptotic or anti-apoptotic genes. Moreover, CHOP-induced apoptosis in ER stress has significant implications for cancers. Most cancer cells present a higher avidity for glucose than normal cells. This depends on a reduced intake of oxygen and nutrients due to inadequate vascularization and diffusion during tumor growth, which, through stabilization of the hypoxia-inducible transcription factor HIF-1α, shifts the cell metabolism toward glycolysis. Glucose shortage associated with cancer cell growth causes ER stress. In fact, ER is the major site for folding and maturation of secretory and transmembrane proteins and requires glucose for protein translation, and for protein glycosylation and folding. ER stress activates a signal transduction pathway termed unfolded protein response (UPR) that induces changes in gene expression to restore ER homeostasis. However, if ER stress cannot be alleviated, UPR triggers apoptosis through PKR-like endoplasmic reticulum kinase (PERK) induction of CHOP. This pathway seems to have an important role in preventing malignant progression [99]. In fact, hepatocyte-specific CHOP ablation increases tumorigenesis in high-fat diet-induced steatohepatitis and HCC [100]. Moreover, deletion of CHOP in a mouse model of K-ras^G12V^-induced lung cancer increases tumor incidence, supporting the tumor suppressor role of CHOP [99].

CHOP triggers the apoptotic pathway through inhibition of BCL-2 and upregulation of BIM and PUMA [101,102], which cause a BAX–BAK dimerization and release of cytochrome *c* from mitochondrial outer membrane. CHOP also induces the expression of DR5, a member of the cell-surface TNF family, which triggers caspase 8-induced apoptosis [103]. Moreover, CHOP can form heterodimers with the transcription factor ATF4 (activating transcription factor 4). These heterodimers increase the transcription of genes involved in protein synthesis, such as *Gadd34, Trb3, Atf3, and Wars*. The increase in protein synthesis causes the depletion of ATP and the oxidative stress that leads to cell apoptosis [104]. Finally, CHOP can suppress the transcription of p21, a cell cycle checkpoint regulator with strong anti-apoptotic activity [105].

CHOP prevents leukemogenesis by activating the PERK–eIF2α–ATF4–CHOP–GADD34 signaling-induced apoptosis in a human hematopoietic stem cell (HSC) pool, with oncogenic mutations [106]. CHOP induction triggers apoptosis of premalignant cells to prevent malignant progression in a mouse lung cancer model [99]. Hepatocyte-specific CHOP ablation increased tumorigenesis in high-fat diet-induced steatohepatitis and HCC. This effect indicates a tumor-suppressive role of CHOP, perhaps via apoptosis of initiated hepatocytes in preneoplastic lesions [100].

However, depending on the cell type, CHOP can show tumor-supporting functions and tumor-induced tolerance. In fact, CHOP acts as an oncoprotein inducing metastasis via transcriptional induction of tumor-associated proteases, both in liposarcoma and fibrosarcoma cell lines and in in vivo models [107]. Moreover, CHOP is upregulated in different mouse models of HCC, as well as human hepatocellular carcinoma. CHOP−/− mice were protected from N-diethylnitrosamine-induced oncogenesis in liver [108]. The number of macrophages and levels of IFNγ and macrophage inflammatory protein-1β (CCL4) mRNA, were markedly reduced in tumors from CHOP KO mice as compared to wild-type mice, suggesting a role for CHOP in modulating the tumor microenvironment and macrophage recruitment to the tumor [108].

CHOP seems to have a critical role in the immune inhibitory activity of tumor-infiltrating myeloid-derived suppressor cells (MDSCs). MDSCs lacking CHOP have decreased immune-regulatory functions and show the ability to prime T cell function and induce antitumor responses. CHOP expression in MDSCs was induced by tumor-linked reactive oxygen and nitrogen species and regulated by the activating-transcription factor-4. CHOP-deficient MDSCs displayed reduced signaling through C/EBPβ, leading to a decreased production of IL-6, and a low expression of phospho-STAT3, whereas the IL-6 expression was sufficient to rescue the immunosuppressive functions of CHOP-deficient MDSCs and restore tumor growth [109]. These data indicate an important role of CHOP in tumor tolerance and potential benefits of its inhibition for tumor immunotherapy.

CHOP seems to be involved in the progression of prostate cancer associated with SPOP mutations (110). SPOP (speckle-type POZ protein) was identified as one of the most frequently affected genes through somatic point mutations in prostate cancer, suggesting SPOP is potentially a key driver for prostate cancer development and progression. SPOP acts as an adaptor protein of the CUL3-RBX1 E3 ubiquitin ligase complex and selectively recruits substrates for their ubiquitination and subsequent degradation. CHOP is a substrate for the SPOP–CUL3–RBX1 E3 ubiquitin ligase complex. SPOP recognizes a Ser/Thr-rich degron in the transactivation domain of CHOP and triggers CHOP degradation via the ubiquitin–proteasome pathway. Therefore, SPOP mutations fail to mediate CHOP degradation, indicating a CHOP involvement in the progression of prostate cancer associated with SPOP mutations [110].

CHOP expression increases dendritic cell expression of IL-23 [111], which supports T helper 17 cell propagation and IL-17 production. IL-17 has been shown to be elevated in several types of cancer. IL-17 induces IL-6 production, which in turn activates oncogenic STAT3, up-regulating prosurvival and proangiogenic genes [112]. The Th17 response can thus promote tumor growth, in part via an IL-6-Stat3 pathway.

Finally, evidences indicate that CHOP can promote specific oncogenic processes when fused with FUS/TLS (fused in sarcoma/translated in liposarcoma) or EWS (Ewing sarcoma) proteins through genomic rearrangement [113,114]. In fact, round cell myxoid liposarcoma (LPS), initially thought to represent a distinct type of liposarcoma, shows the same t(12;16)(q13;p11) translocations and represents a high-grade form of myxoid LPS. The translocation results in fusion of the CHOP gene in 12q13 and the FUS/TLS gene in 16p11. Moreover, the fusion of the N-terminal part of the EWS gene with the CHOP gene, as a result of recombination of the 12q13 and 22q11–12 bands, was also shown in high-grade forms of myxoid LPS.

## 5. Conclusions

C/EBPs have a pivotal role in numerous cellular functions, including proliferation, differentiation, apoptosis, and ER stress. Moreover, C/EBPs are implicated in a number of diseases including cancer. Although C/EBPs prevalently display an oncosuppressive activity, the ability of these transcription factors to act as tumor promoter in specific conditions and depending on the type of tumor should be considered when new natural or synthetic compounds that are able to modulate their activity are developed for cancer therapy. Indeed, several in vitro studies have shown that molecules that increase the expression/activity of different C/EBPs can inhibit cell growth and cancer progression, promoting apoptosis in tumor cells through different pathways [115,116,117,118,119]. However, for the “Janus” role that C/EBPs have shown in cancer progression, an extensive in vivo experimentation should be carried out for any compound considered for C/EBP modulation. Finally, further studies are needed to better understand the molecular mechanisms underlying the diverse functions of the different isoforms of C/EBPs and their role in cancer progression.

## Figures and Tables

**Table 1:** Tissue distribution of the various members of the C/EBP family.

C/EBPα: adipose tissue, blood mononuclear cells, liver, intestine, lung, adrenal gland, blood, nervous system, and placenta.
C/EBPβ: liver, adipose tissue, myelomonocytic cells, intestine, lung, spleen, kidney, and nervous system.
C/EBPδ: adipose tissue, myeloid cells, lung, intestine, and nervous system.
C/EBPε: myeloid and lymphoid cells.
C/EBPγ: ubiquitous expression.
CHOP: ubiquitous expression.

**Table 2:** Molecular mechanisms of tumor suppressor and the tumor promoting activity of each C/EBP family member.

C/EBP Type	Tumor Suppressor Activity	Tumor Promoting Activity	References
C/EBPα	Phosphorylated form at Ser 190 (193).	Dephosphorylated form at Ser 190 (193). Mutation of Ser 193 to Ala.	[39,40]
C/EBPβ	Phosphorylated isoform.	Dephosphorylated isoform.	[58,59,60,61,62,63,64,65]
	β:β homodimers.	β:γ heterodimers.	
	Compartmentalization in perinuclear cytoplasm.	Compartmentalization in peripheral cytoplasm.	
	Low LIP/LAP ratios.	High LIP/LAP ratios.	
C/EBPδ	Downregulation of cyclin D/E, C-Myc and upregulation of P27CIP2 in the early stages of tumor development.	Increasing translational activity of HIF-1α in breast cancer metastasis. Overexpression of HIF-1 α and downregulation of FBXW7α in glioblastoma. Overexpression of IGF-1 and PDGFA-R in cultured osteoblasts.	[54,66,71,78,79,80,83]
C/EBPγ	Inability to suppress C/EBP-mediated growth arrest in hepatoma cells. Inability to suppress C/EBPα growth arrest in different cell lines.	Inhibition of cellular senescence through heterodimerization with C/EBPβ.	[90,91]
C/EBPε	C/EBP-ε^32^ and C/EBP-ε^30^ isoforms are transcriptional activators that cause exclusively eosinophil differentiation. No specific effects on cancer.	C/EBP-ϵ^27^ is an inhibitor of GATA-1 inhibits eosinophil differentiation promoting granulocyte-macrophage differentiation. C/EBP-ε acts as a dominant-negative regulator. No specific effects on cancer.	[92,93,94]
CHOP	Induction of apoptosis by inhibition of Bcl-2 and upregulation of Bim, PUMA, DR5 and p21.	Activation of MDSCs. TH17 propagation that promotes tumor growth via IL6-STAT3 pathway. Fusion with FUS/TLS or EWS by genomic rearrangement.	[98,99,100,101,102,106,108,109,110,111]
